# The Role of Perspective Taking on Attention: A Review of the Special Issue on the Reflexive Attentional Shift Phenomenon

**DOI:** 10.3390/vision3040052

**Published:** 2019-10-09

**Authors:** Gabriele Pesimena, Christopher J. Wilson, Marco Bertamini, Alessandro Soranzo

**Affiliations:** 1 Department of Psychology Politics and Sociology, Sheffield Hallam University, Sheffield S10 2BP, UK; dsas2@exchange.shu.ac.uk; 2 School of Social Sciences, Humanities & Law, Teesside University, Middlesbrough TS1 3BX, UK; Christopher.Wilson@tees.ac.uk; 3 Department of Psychological Science, Liverpool University, Liverpool L69 3BX, UK; M.Bertamini@liverpool.ac.uk; 4 Department of General Psychology, University of Padova, 35131 Padova, Italy

**Keywords:** reflexive attentional shift, visual attention, alter-centric intrusion, viewpoint, gaze perception, theory of mind, perspective taking

## Abstract

Attention is a process that alters how cognitive resources are allocated, and it allows individuals to efficiently process information at the attended location. The presence of visual or auditory cues in the environment can direct the focus of attention toward certain stimuli even if the cued stimuli are not the individual’s primary target. Samson et al. demonstrated that seeing another person in the scene (i.e., a person-like cue) caused a delay in responding to target stimuli not visible to that person: “alter-centric intrusion.” This phenomenon, they argue, is dependent upon the fact that the cue used resembled a person as opposed to a more generic directional indicator. The characteristics of the cue are the core of the debate of this special issue. Some maintain that the perceptual-directional characteristics of the cue are sufficient to generate the bias while others argue that the cuing is stronger when the cue has social characteristics (relates to what another individual can perceive). The research contained in this issue confirms that human attention is biased by the presence of a directional cue. We discuss and compare the different studies. The pattern that emerges seems to suggest that the social relevance of the cue is necessary in some contexts but not in others, depending on the cognitive demand of the experimental task. One possibility is that the social mechanisms are involved in perspective taking when the task is cognitively demanding, while they may not play a role in automatic attention allocation.

A towel, it says, is about the most massively useful thing an interstellar hitchhiker can have [..] wrap it around your head to avoid the gaze of the Ravenous Bugblatter Beast of Traal (a mind-bogglingly stupid animal, it assumes that if you can’t see it, he can’t see you—daft as a brush, but very very ravenous).

Douglas Adams

## 1. Introduction

The Ravenous Bugblatter Beast of Traal in Douglas Adams’s *The Hitchhiker’s Guide to the Galaxy* [1] is a beast so stupid that it thinks that if a person cannot see it, then it cannot see that person. Therefore, we can cover our eyes with a towel to avoid being attacked. Here, Douglas Adams is playing with the idea that people are aware that they might be biased to favour their own view, and he surprises the reader by offering a reverse scenario. However, it is possible that people are a little bit like the Ravenous Bugblatter Beast of Traal in that they cannot help being affected by what other people see. This is the key tenet of a social perspective-taking view of attention.

Attention can be oriented by different cues, such as arrows [2] or eyes [3]. These cues can produce an automatic (or reflexive) rather than a voluntary orientation of attention [4].While voluntary shift depends upon the observer’s expectations and intentions, reflexive shifts of attention (RAS) are associated with sensory stimulation and generated by unforeseen changes in the visual field, particularly by the abrupt onset of stimuli, which elicit reorienting and saccadic eye movements [2].

**Figure 1:**
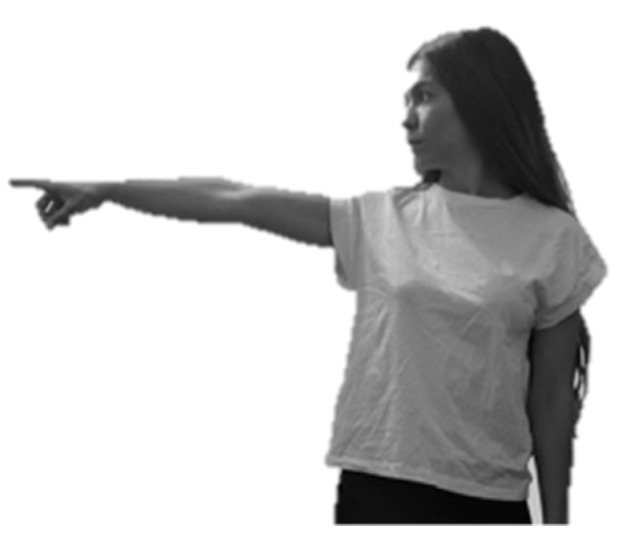
Our attention is reflexively shifted toward where this person is looking and pointing.

In particular, taking in consideration the notion of “Theory of Mind” [5], it has been suggested that attention can be reflexively shiftedtoward where another person is looking (Figure 1), which causes errors or slower responses when reporting what we see, if this is different from what the other person sees [6].

**Figure 2:**
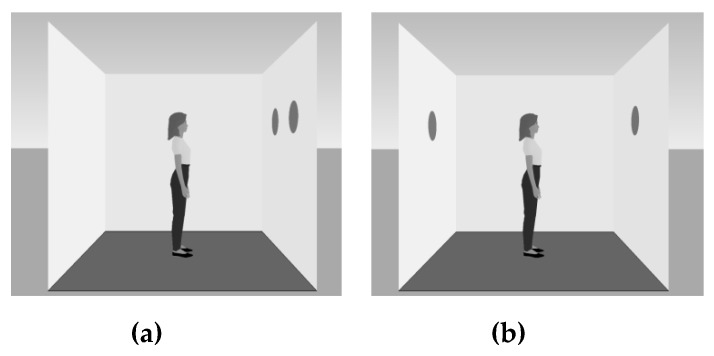
An example of consistent (**a**) and inconsistent (**b**) trials in the dot perspective task.

To investigate the Reflexive Attentional Shift (RAS) phenomenon, Samson et al. [7] devised a semi-experimental paradigm. The general setup consists of a 3D virtual room presented on a computer screen with the back, left, and right walls visible. A human avatar placed in the center of the room is used as a cue to direct attention toward either the left or right wall of the room. During the experiment, discs appear on either the left, right, or both walls. The participants’ task is to indicate (in each trial) how many discs they or the human avatar can see. As the participants see both the left and right walls, they can see all of the discs. However, since the human avatar faces left or right, it can only see the discs placed on one side. Therefore, there are consistent and inconsistent trials. In consistent trials, the number of discs visible to the participant and to the avatar is the same. In inconsistent trials, the participant can see some discs that the human avatar cannot (Figure 2). Participants respond as quickly as possible, with responses >2000 ms counted as errors. Reaction Times (RTs) and errors are the dependent variables. 

The authors confirmed the existence of egocentric bias: reporting what the avatar can see is affected by what the participant can see. However, they also found interference (i.e., longer reaction times and more errors) in inconsistent trials even when participants had to report how many disks they can see. This interference is defined as “alter-centric intrusion.” Various egocentric biases are known and have been extensively studied such as Piaget’s three mountain task [8]. An alter-centric bias is, by contrast, a less well-documented phenomenon. Both can, in theory, co-exist.

To explain the alter-centric intrusion effect, Samson et al. [7] advanced the “Perspective-Taking” theory, asserting that people spontaneously incorporate the viewpoint of others. Since then, several studies have reported supporting evidence for this theory [9,10,11].

Nuku and Bekkering [11], Teufel et al. [12], and Furlanetto et al. [13] found that alter-centric intrusion is present when participants are asked to judge their own perspective while the human avatar was believed to be able to see. On the other hand, intrusion was not present, when the human avatar was believed to be unable to see (e.g., its line of sight obscured).

These findings, supporting the Perspective-Taking theory, run counter to the “Perceptual” theory, which argues that the perceptual features (i.e., the direction of other’s face/nose/posture) are sufficient to explain attentional orientation [14,15,16]. These studies found that human avatars spontaneously orient attention of the observers even when the human avatars cannot see the stimuli either because a physical barrier prevents the view, as in Cole et al. [14] or when the cue employed in the dot perspective task does not have a mental state (e.g., an arrow, or a camera) as in Wilson et al. [16]. According to this theory, the effects showed in the dot perspective task are due to domain-general processes rather than perspective taking.

In an effort to bridge this gap and clarify the mechanism behind social perception, Michael and D’Ausilio [17] suggested that the dot perspective task itself may engage both Theory of Mind and domain-general processes. Social and non-social clues would, therefore, engage the same attentional process eliciting RAS, despite being represented by two different functional systems.

Further research has focused on whether the perspective may not be a spontaneous phenomenon. If this is the case, then RAS would be only modulated by perceptual characteristics of the cue, while perspective taking would be due to top-down processes [18,19,20,21]. These authors noted that the human avatar employed by Samson et al. [7] was unable to generate an attentional shift when the target discs were presented within 300 ms from the presentation of the cue. The authors, therefore, concluded that attentional shift may be induced by taking a perspective, but this cannot be defined “reflexive” since it requires some time to occur. 

Inspired by the previously mentioned literature in which similar results may be interpreted either in agreement with the perceptual characteristics of the cue (perceptual position) or its social characteristics (social position), the journal *Vision* recently hosted a special issue titled “Reflexive Shifts in Visual Attention.” This special issue (www.mdpi.com/journal/vision/special_issues/RAS) provided a place where some of those studies, supporting old and new theories behind RAS, are collated. This review is, therefore, intended as an overview of contemporary research on the RAS phenomenon, which summarizes each of the contributions to this special issue on RAS and briefly outlines directions for future research.

## 2. Visual Attention and Reflexive Attentional Shift

We are constantly surrounded by a world containing more information and objects than what our cognitive system can process. Attention allows us to choose and select certain stimuli and ignore others. The complexities of attention are shown by neuroimaging data illustrating how attention is carried out by a network of anatomical areas and is, therefore, neither the property of a specific brain region nor a function of the brain as a whole [2]. In particular, it has been shown that the existence of three networks is related to a different aspect of attention, which alerts orienting and executive controls [2,22]. As pointed out by Carrasco [22], attention seems to be influenced and facilitated by previous knowledge and assumption of the surrounding world. This places attentional processes halfway between perception and cognition. Our attention can, therefore, be influenced by different factors, which can be grouped in two main categories: bottom-up (or exogenous) factors, in which attention is usually deployed reflexively due to the characteristics of the scene and stimuli’s salience, and top-down (or endogenous) factors, in which attention is often deployed voluntarily in accordance with specific tasks or goals, and with the task or goal having a strong influence on where the participants allocate their attention.

In her contribution to this special issue, Zhaoping [23] provided further insights on the mechanisms behind visual attention orientation. Previous results showed that a target stimulus is localised quicker if it is presented to only one eye [24]. The author investigated whether the ocularity contrast of a visual input, which is a feature that is often hardly visible, captures attention exogenously, where ocularity of a visual input refers to the difference of visual input between the two eyes. Results from the study showed that, regardless of its task relevance, a visual location with a strong ocularity contrast attracts attention. These findings are in line with previous literature, which supports the idea that the primary visual cortex creates a bottom-up saliency map to guide attention exogenously. According to these studies, target characteristics, such as changes in luminance, motion, or color, are combined in a spatial map, which highlights the most salient aspect, as a consequence of which attention is reflexively shifted [25,26].

Extending Zhaoping’s study [23], Burnett et al. [27] examined specific characteristics of exogenous cues that are either more or less likely to draw attention. They used a dual-task paradigm to test whether luminance or an equiluminant colour change modulated motion and colour discrimination effects. Their results showed that the motion and colour tasks were affected differently by the two cues. Motion validity was more strongly affected by luminance than colour cues, whereas the colour validity showed no difference in effect between luminance and colour cues. These results have implications for our understanding of how low-level properties of cues could influence visual attention, with the authors suggesting that “cues which engage the same visual channel as the target are more effective in enhancing target processing at the cued location.” Moreover, if further work supports this view that exogenous cueing is not a unitary process, then this will need to be considered when studies apply cueing tasks.

## 3. Reflexive Attentional Shifts: Perceptual or Social Mechanisms?

The fact that attention can be reflexively oriented by the incidental information provided by cues and contextual information provided during a cueing task is the basis for one of the core debates examined in this special issue. This includes whether reflexive biases in attention are modulated by social or perceptual processes. While there is a body of evidence showing that attention can be reflexively biased toward where another person is looking, which causes errors or slower responses when reporting what we see if it is different from what the other person sees [6], there is no consensus within the literature on what causes this attentional shift. The contributions to this special issue attempt to clarify the factors involved in the RAS phenomenon and, as a result, provide evidence in support of one or another interpretation. 

### 3.1. Perceptual Interpretations of RAS

**Figure 3:**
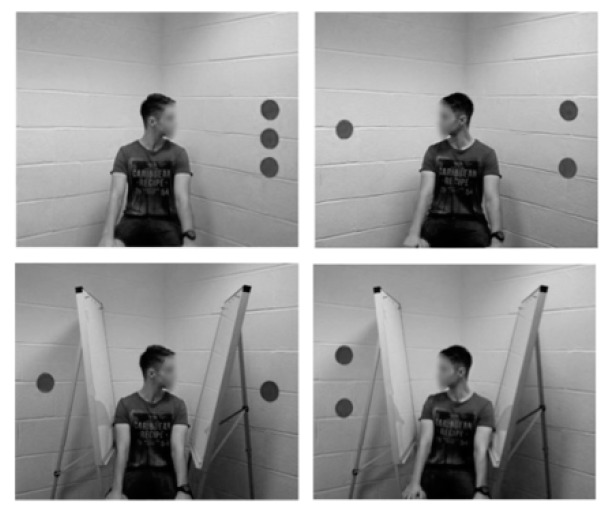
Example of the stimuli used by Langton [28].

Langton [28] hypothesized that the inconsistent results between perceptual and social interpretation could be due to the use as a directional cue of a computer-generated avatar, or a photograph of a person, rather than a real person. While it can be expected that an avatar (or a photograph of a person) generates orienting effects, this does not necessarily mean that there is a spontaneous attribution of visual perspective. Thus, Langton’s work [28] is in line with Wiese et al.’s [29] and Gardner et al.’s [21] studies, which suggest that participants must believe that the avatar represents an intentional agent in order to take its point of view. If intentionality is not attributed to the cue, then its directional effects are due to its perceptual features. Langton [28] set up two experiments both employing the dot perspective paradigm [7] but replacing the avatar with photo-realistic stimuli in experiment 1, and with real persons in experiment 2 (Figure 3).

Both experiments tested two main conditions: seeing the condition in which the gazer was able to see either the screen at its left or its right, and the non-seeing condition, in which barriers were placed between the gazer and the screens.

Hence, Langton’s results [28] support the hypothesis that the interference found in previous studies may be due to the perceptual feature of the directional cues, not from an implicit mentalizing of the other’s perspective. 

**Figure 4:**
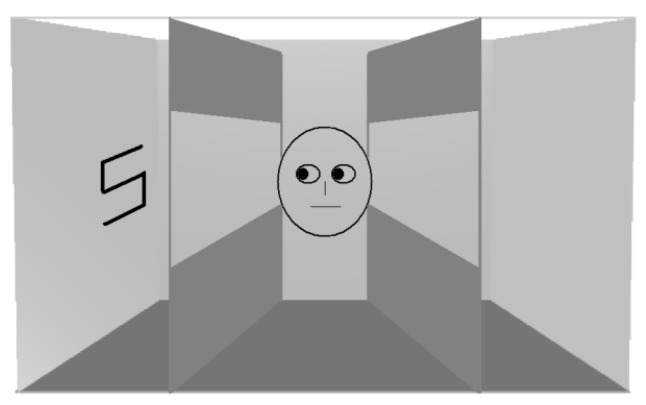
One of the stimuli employed by Cole et al. [19].

Participants were, in fact, faster on consistent trials (the number of target dots visible to participants equal to the number of dots visible to the gazer) rather than inconsistent trials (where the number of dots visible to the gazer differ from the number of dots visible to the participant) in both a seeing and non-seeing condition in the two experiments. On another contribution to this special issue, Cole et al.’s findings [19] supported Langton’s results [28] in favour of the “Perceptual theory.” Cole et al. [19] pointed out how the choice of specific tasks and cues may influence the alter-centric intrusion phenomenon. In their work, Cole et al. [19] argued that, to attribute RAS to social factors, the interference should take place in all settings in which the avatar sees the same stimuli as the participants. To test this assumption, the authors incorporated the presence of an avatar within two other classic visual cognition tests. Regardless of whether the avatar could or could not see the same things of the participants, no cuing effects emerged, which shows that an avatar cannot generate reflexive directional shifts in all task types. Directional cueing effects emerged in the authors’ study only when more perceptually salient cues were used, such as a schematic representation of a face (Figure 4).

Overall, Cole et al. [19] showed that the “perspective taking” effect observed in the dot perspective task does not generalize to other paradigms. Cole et al.’s results [19], therefore, supported Gardner’s position [21] that the avatar itself it is not sufficient to generate spontaneous attribution of visual perspective at least when the target is presented within a short interval after the avatar. 

The abovementioned studies seem to point toward a theory, in which the chosen tasks, stimuli, and the relative cognitive demands, which will be shown later, may play a key role in generating the RAS phenomenon. This interpretation seems to be confirmed by Albonico et al. [30] and Kulke’s [31] contributions to this special issue, which underlined the importance of choosing the appropriate set of tasks and stimuli when the aim is to measure an attentional shift.

Albonico et al. [30] provided evidence that the deployment of focal attention depends on the interaction between the task demand and the type of stimuli. Furthermore, they show that this process is mainly reflexive. The authors measured focal attention by means of the cue-size effect magnitude, which is the inverse relationship between the size of the focus of attention and the concentration of resources within the attentional field. For the task demand, the authors compared detection with discrimination tasks, in which the former requires fewer attentional resources than the latter. For the stimuli type, the authors compared high-level representational stimuli (letters) with low-level representational stimuli (geometric shapes). Furthermore, the authors manipulated the Stimulus Onset Asynchrony (SOA) between a cueing and a target stimulus. Results show that high-level representational stimuli elicit a larger cue-size effect than low-level representational stimuli when the task is a detection task, while there is no difference between the two types of stimuli in the discrimination task. These findings may explain the different findings in the literature on the deployment of focal attention. A discrimination task may not show the differences between high-level and low-level representational stimuli. In addition, considering the temporal delay between cueing stimuli and the target, the authors found that the cue-size effect enhances at short SOAs. This suggests that focal attention is mainly a reflexive process.

In line with Albonico et al. [30], Kulke [31] pointed out the importance of comparing the results of different paradigms (the fixation shift paradigm and the gap-overlap paradigm) used to measure an attentional shift between stimuli that are often used as predictors for developmental outcomes. Hence, Kulke [29] investigated the effect of eccentricity (defined as the angular distance from the center of the visual field) and target size on attentional shift latencies (the time taken from the appearance of a target to the beginning of a saccade in response to that target). The author systematically manipulated the target’s eccentricity (great and small eccentricity) and size (big size and small size) measuring the potential differences in eye-movements’ responses to stimuli of different sizes and eccentricities within the two previously mentioned paradigms. Results showed that eccentricity and target size affected the attentional shift. Subjects responded more slowly to the big target stimuli when it was closer to the center of the screen (big target size, small eccentricity) and vice-versa (small target size, great eccentricity). However, no significant differences in refixation latency between targets were found when the target stimuli’s size was scaled in proportion to the eccentricity. This is the case of the fixation shift paradigm (big size, great eccentricity) and of the gap-overlap paradigm (small size, small eccentricity). The author, therefore, concluded that the results recorded in experiments based on the fixation shift paradigm and on the gap-overlap paradigm may be compared as long as the stimulus size is scaled in proportion to their eccentricity.

### 3.2. Social Interpretations of RAS

The studies reviewed explain the RAS phenomenon in terms of the perceptual features of the directional cue. The following reviewed studies published in the special issue support the social interpretation. Morgan et al. [32] focused on the importance of attributing a mental state to a human avatar employed as a cue. In particular, they tested whether the avatar’s gaze mediates the shift of attention. Teufel et al. [12] showed that, when the human avatar is not believed to be capable of seeing, it does not interfere with our attention. While, when the human avatar is perceived as a “viewer,” its gaze does affect our attention. Although Morgan et al. [32] share with Teufel et al. [12] the same position, they underlined that Teufel et al.’s work [12] presented a number of weaknesses that could limit the interpretation in favour of the social account of RAS. Hence, Morgan et al. [30] specifically noted that Teufel et al. [12] (1) used a response time task. This method may not be sensitive enough to detect subtle attentional shifts. (2) This method employed a blocked design. The two conditions of viewing and not-viewing were in blocks instead of interleaved. This experimental design could have led participants to suppress orienting in response when the Other was not been able to see and (3) encouraged the attribution of a mental state to the Other by providing leading instructions.

**Figure 5:**
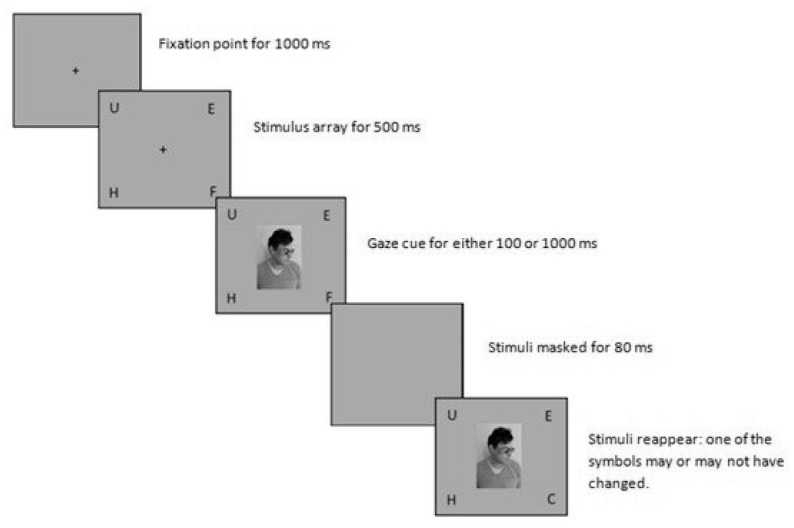
The experimental procedure employed by Morgan et al. [32].

In their instructions, Teufel et al. [12] encouraged the participants to take the viewpoint of the Other. In this way, participants may have believed that they were expected to answer differently to the two conditions. To overcome the above potential problems, Morgan et al. [32] (Figure 5) (1) used a change detection paradigm, (2) interleaved trials between conditions, and (3) did not provide instructions to the participants. 

While controlling for these potential confounding variables, Morgan et al.’s results [32] were in line with Teufel et al.’s [12]. Participants were influenced by the Other’s gaze only when s/he was able to see. However, Morgan et al. acknowledge that their results are not consistent with similar studies conducted by Cole et al. [14,19], which showed that the mental state does not influence attentional orienting. The reason for different results is not clear yet. However, this can be due to the use of different paradigms and stimuli manipulation adopted within each study, in order to test the different mental state conditions.

**Figure 6:**
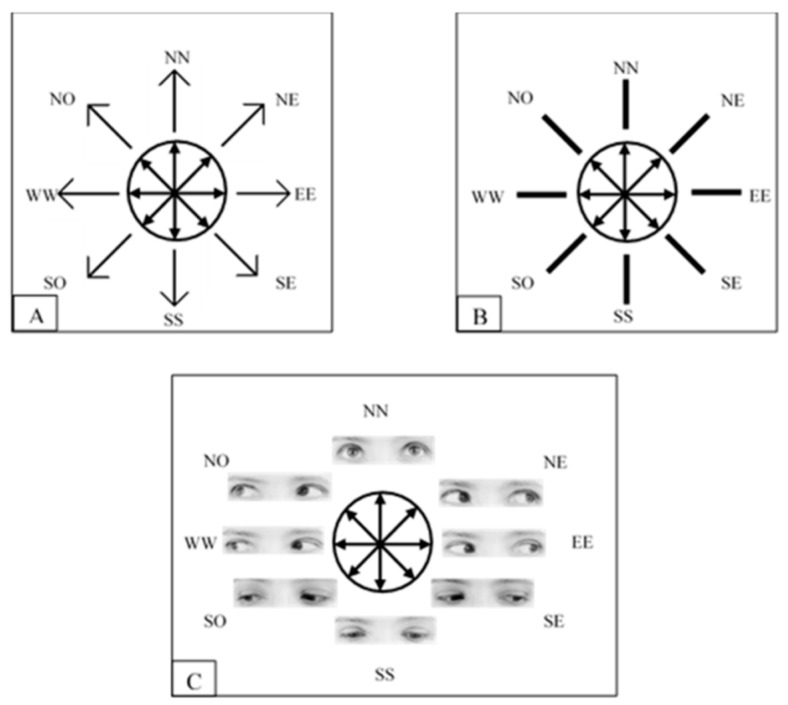
The three types of stimuli used in Actis-Grosso and Ricciardelli [33]. Each panel displays the eight directions conveyed by the different stimuli and the eight possible positions of stimulus presentation.

In their contribution to this special issue, Actis-Grosso and Ricciardelli [33] highlighted the role of social factors in generating RAS. The authors tested whether stimuli known to automatically orient visual attention, such as arrows and averted gazes (Figure 6), also modulate the correspondence problem, which is the problem of ascertaining to which objects in one frame correspond to the objects presented in a subsequent frame. The authors hypothesized that the stimuli known to trigger RAS should also drive the correspondence problem. The comparison between the arrows and the averted gazes is unique for the purpose of this research. To this end, the authors compared the effects of arrows and averted gazes with those of lines which should be considered as a baseline.

It emerged that all the three types of stimuli generate an RAS effect and that they all modulate a correspondence problem. Furthermore, the effect generated by arrows and gazes is stronger than those generated by lines. Furthermore, it was found that the effects of arrows and averted gazes are equivalent when they are in a comparable condition. However, when there is a directional conflict of information, rather than a weakening, the effect of the gazes becomes stronger than those of arrows. As can be seen in Figure 6, the rectangular boxes, which encompass the gazes, always have a horizontal direction. A directional conflict occurs when the gazes point toward any non-horizontal position.

In line with Morgan et al. [32], Actis-Grosso and Ricciardelli concluded that stimuli known to automatically orient visual attention, such as a gaze direction and arrows, influence the correspondence problem more than lines. Furthermore, gazes are more powerful than arrows in generating RAS when there is a spatial conflict.

**Figure 7:**
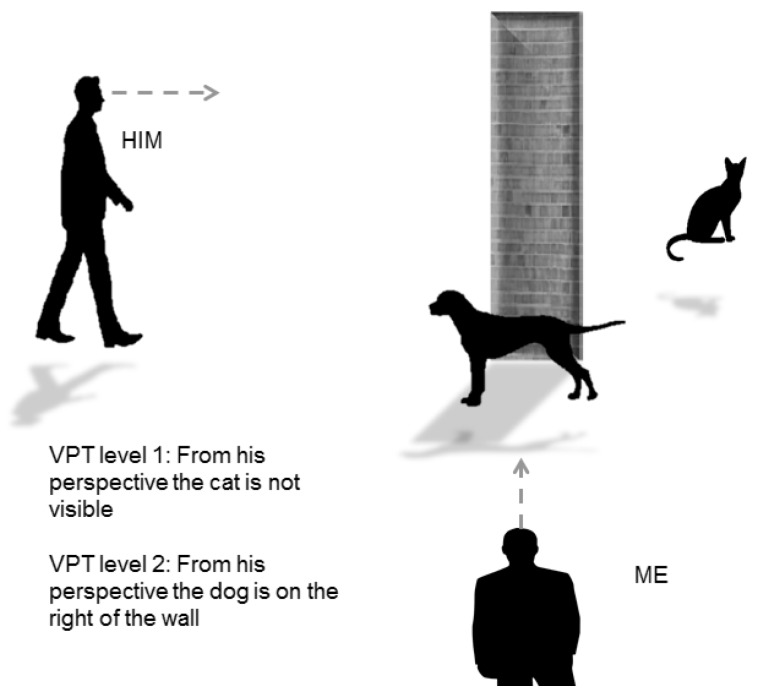
Visual Perspective Taking (VPT) level 1 and Visual Perspective Taking (VPT) level 2.

The role of social mechanisms and perspective taking in generating RAS was also supported by Gardner et al. [34]. The authors examined assumptions inherent in the sub-mentalizing account of the altercentric intrusion phenomenon during level 1 Visual Perspective Taking (VPT). VPT is defined as the ability to understand that other people have a different line of sight from us, whereas the VPT level 2 is the understanding that two people viewing the same item from different points in space may see different things [35,36] (Figure 7). 

**Figure 8:**
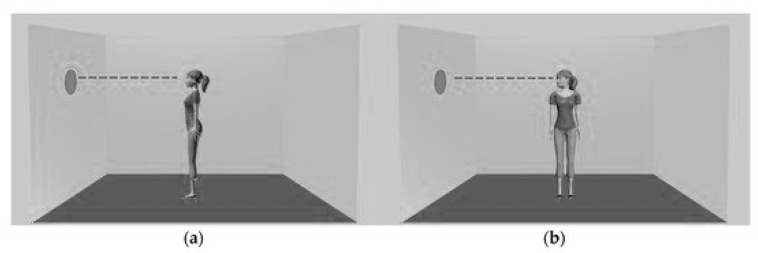
Gaze-maintained and gaze-averted avatars used by Gardner et al. [34].

Specifically, the researchers manipulated cue stimuli in a way that aimed to influence visuospatial attentional orienting but not mentalising. Specifically, they presented avatar cues in two positions: gaze-maintained avatars where body position was consistent with gaze direction and gaze-averted avatars where the body position was perpendicular to a gaze direction (Figure 8).

Their first experiment presents several unique findings including some of which are consistent with previous work and others that are less expected. First, the finding that attention orienting was present only for longer SOAs indicates that attentional orienting might not be reflexive. Second and perhaps more pertinent to the focus of this study, the gaze-averted cues showed an effect of validity on (RTs). Participants were faster when the target appeared at the cued location compared to the non-cued locations. There was, instead, no effect of validity on RT when the gaze-maintained cues were employed. In a second experiment, they examined the difference in reaction times between consistent and inconsistent trials, and found no effect for the avatar stance. They take this finding as evidence that cue features (such as gaze/stance orientation) that influence attentional orienting do not necessarily affect level 1 visual perspective-taking. The potential dissociation between perspective-taking and attentional orienting has important implications for both implicit mentalising and sub-mentalising accounts that have been put forward to explain this phenomenon.

## 4. Additional Factors Involved in RAS

Further contributions to this special issue, rather than focusing on the social-perceptual debate, placed their focus on the different variables that may affect and influence RAS and the attentional cueing paradigms such as temporal information, changes in tonic alertness, and inter-individual differences. 

### 4.1. The Influence of Temporal and Auditory Information

Among those contributions, Laidlaw and Kingston [37] investigated how ignoring temporal information eliminates reflexive spatial orienting. In particular, the authors investigated whether the interaction between temporal and spatial attention modulates the Reflexive Attentional Shift. Temporal attention refers to the process of allocating brain resources on the predicted onset of an incoming event [38]. To investigate this interaction, the authors explored the fore period effect [39]. This is the effect by which the cuing of a target generates an inverse relationship between subjects’ reaction times and the time between the cue and target appearance: longer time between the stimuli results in shorter reaction time.

The authors systematically manipulated spatial characteristics of the cue (arrows-to elicit reflexive attention versus letters-to elicit volitionally attention), SOA (100, 500, and 1000 ms) and congruency of the cue (congruent versus incongruent). 

The results showed the emergence of a fore period effect and of a spatial cueing effect with both arrows and letters, but only at longer SOAs and only in congruent conditions. On the other hand, with shorter SOAs and in incongruent conditions, the fore period effect did not occur while the spatial cuing effect occurred only with letters. 

The authors, therefore, concluded that only reflexive spatial attention orienting is modulated by the implicit changes in temporal attention, while volitional spatial attention is not. Thus, the way in which spatial and temporal attention interact must be taken into serious consideration during visual attentional studies.

**Figure 9:**
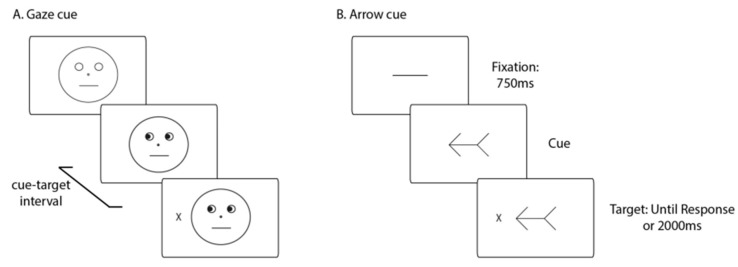
Example of cues and task sequence employed by Hayward and Ristic [40].

Extending Laidlaw and Kingston’s research [37], Hayward and Ristic [40] investigated two different processes that may be present in any study involving spatial cueing: tonic alertness and voluntary temporal preparation. In this study, the authors tested whether changes in tonic alertness and voluntary temporal preparation affect attentional orienting. They confirmed that a task-relevant social gaze and non-social arrow cues affected spatial attention, with no differences between the two cues (Figure 9).

They found that the magnitude of the generated attentional shift may be modulated by high tonic alertness, while no differences were found with voluntary temporal preparation. Even if, overall, those results seem to be contrasted with Laidlaw and Kingston [37], both studies seem to converge on the idea that the cue generated an attentional shift that appears to remain robust across different cueing task settings. However, the task parameters seem to play an important role when modulating the magnitude of the attentional orienting effect elicited by the different types of cues.

On a similar line of enquiry, Klein [41] focused on the control of visual attention by auditory stimuli. In a series of cross-modal experiments using the cueing paradigm, the author presented to the subjects an auditory cue indicating the position of a target manipulating the cue informative value (congruent versus incongruent with the location of the target) and its onset asynchrony (SOA).

The results showed that the informative value of the auditory cue affected the target localisation. This suggests that localizable auditory stimuli exogenously (rapidly and automatically) capture visual attention. In addition, it was found that subjects were faster to identify the cued target at short SOA, while participants were slower when SOA was between 500 and 1000 ms. The author, therefore, concluded that, for SOA within this temporal window, the exogenous shift of attention is overcome by the endogenous one. 

**Figure 10:**
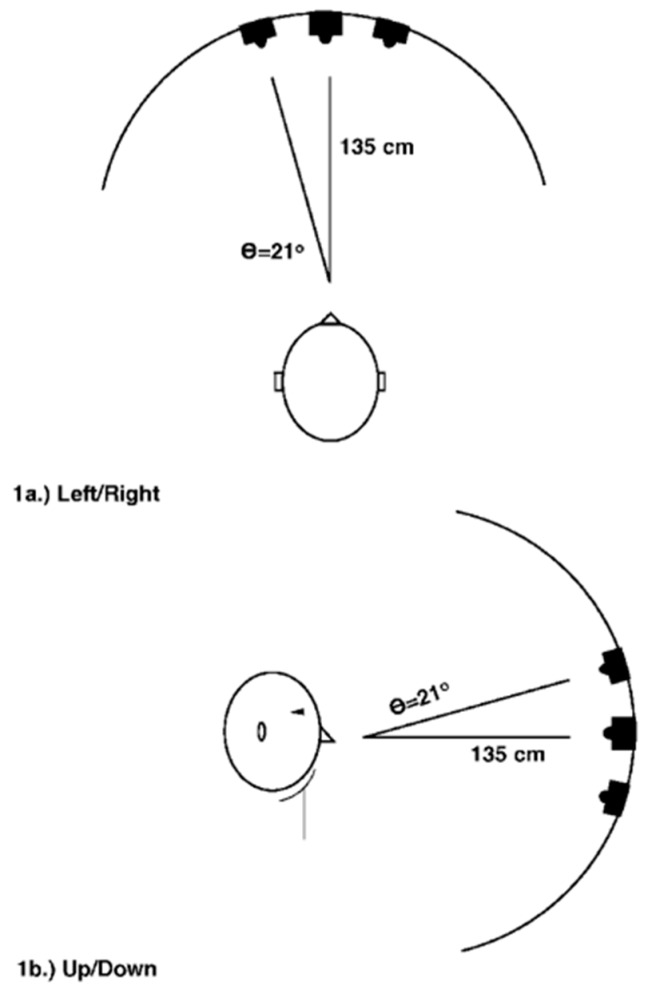
Example of the apparatus employed by Klein [41].

Furthermore, in another series of experiments, the author manipulated the auditory cue changing its pitch rather than its location (Figure 10). The cue was centrally presented but its glide frequency was manipulated to indicate the target position. The glide frequency could have been informative (raising tone indicating top location and vice-versa) or uninformative. Subjects were faster in the informative conditions, which shows that changes in the glide of the auditory cue shift attention only when it is meaningful.

### 4.2. Inter-Individual and Laboratory-Real World Differences

Inter-individual differences and the differences between laboratory settings and the real world are usually overlooked when attentional orienting and/or taking a perspective are investigated. In their contribution to this special issue, Bukowski and Samson [42] explained some of the individual differences in terms of the ability to handle conflict between two conflicting perspectives, and the variability in the strength of the egocentric perspective. The study used a visual perspective-taking task and a large sample. Results showed that individuals varied in their difficulty in considering another person’s differing perspective. A cluster analysis suggested four underlying profiles, which can be placed within a two-dimensional space. The two axes are the ability to handle conflict and the relative attentional focus on the self rather than the other person’s perspective. 

**Figure 11:**
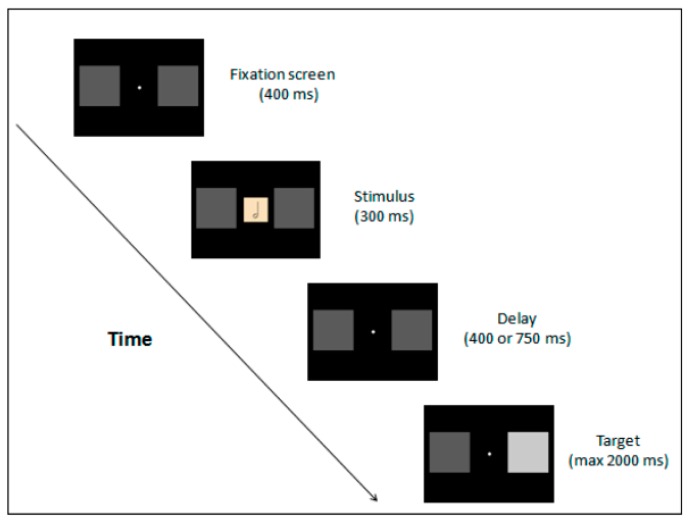
Task sequence employed by Prpic [43]. In this example, the stimulus was the half note and the target appeared on the right visual field.

In line with Bukowski and Samson’s findings [42], another contribution to this special issue seems to highlight the importance of inter-individual differences. Prpic [43] investigated how perceiving musical note values causes a spatial shift of attention in musicians (Figure 11). The author contributed to the discussion on RAS by taking into consideration the Spatial-Numerical Association of Response Codes (SNARK) effect. This is the phenomenon by which perceiving numbers can affect the allocation of spatial attention, which causes a leftward target detection advantage after perceiving small numbers and a rightward advantage for large numbers. The aim of the study was to test whether the effect can be reproduced in musicians when reading musical notes instead of numbers. The visual representation of the duration of musical notes shares with the numbers a symbolic representation that goes from left to right. Specifically, images depicting whole and half notes represent a relatively long duration, while eighth and sixteenth notes represent a short duration.

The author found an advantage in detecting a leftward (vs. rightward) target after perceiving small (vs. large) musical note values, which suggests that musicians process numbers and note values in a similar manner. Future studies on RAS might benefit these findings for testing whether the SNARK is affected by the presence of an “Other” on either side of the stimuli presentation. 

**Figure 12:**
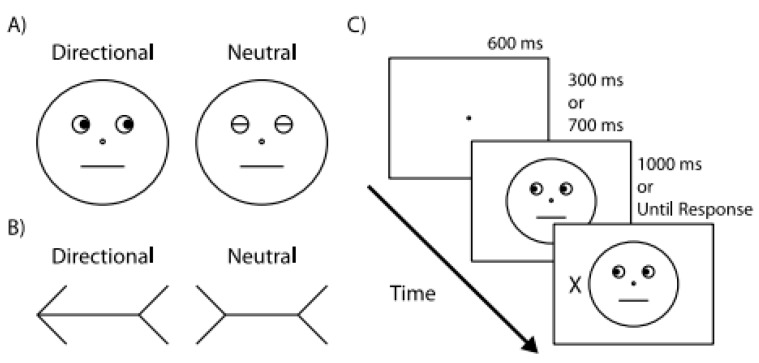
Example of cues and of a valid trial employed by Blair et al. [44].

Lastly, Blair et al. [44] presented a way for assessing individual instances of cover attentional orienting in response to gaze and arrow cues. The authors investigated whether gaze-following behavior occurs in laboratory tasks as frequently as in natural settings. In the first experiment, the presence of costs or benefits in cue trials was calculated, i.e., the proportion of RT responses falling more than 1 SD outside of the performance of neutral control trials. The authors, then, replicated the study in a second experiment with a different directional cue, which serves as the control comparison. The results of both experiments suggest that attentional orienting in gaze-cuing tasks is infrequent, and occurs in less than 50% of trials. However, even though benefits and costs occurred in less than 50% trials, which is consistent with the literature, results indicated that more benefits relative to costs occurred in a valid trial (stimulus appears on targeted location) (Figure 12). More costs relative to benefits occurred in invalid trials (stimulus appears on non-targeted location). Furthermore, the results showed no differences between gaze cues and arrow cues.

These results have important implications for the use of cueing tasks in the lab. The theoretical explanations that come from their use and the analysis method employed presents a useful starting point for examining the frequency of attentional orientation in future gaze-cueing studies within and across real world and laboratory investigations.

## 5. Conclusions and Future Directions

On one hand, observers are good at knowing where another person is looking [45]. On the other hand, there are also limits and mistakes in reasoning about the role of a viewpoint in a scene [46]. This paper focused on how attention is affected by the presence of another individual in the scene. Researchers have shown that this other individual may act as a cue directing our attention. However, there is no agreement on how this process works.

Some research studies show that the other individual has the same role as any other directional cues that can bias attention such as an arrow. Other research studies, however, show that observers are specifically sensitive to the social characteristics of the other individual, and, therefore, are affected by the content of another person’s viewpoint. In this review, we consider the contributions that appeared in the special issue on Reflexive Attentional Shift (RAS) published in *Vision*. Establishing whether RAS is a perceptual or a social process is important because RAS is used as a measure of visual perspectives and mental state attribution in both developmental and clinical contexts. For example, visual perspectives may be used to evaluate children development with regard to the Autism Spectrum Disorder (ASD) [36,47,48]. The contributions of this special issue allow the reader to reach deeper insights into the RAS phenomenon, by not only focusing on the importance of understanding the nature of the process behind it, but also providing further theories and knowledge about different variables that may influence or elicit RAS. 

Taking all evidence into account, the contributions confirm that human attention is biased by the presence of a directional cue in the scene. By analyzing the different experiments, it appears that the social relevance of the cue may be necessary in some contexts but not in others.

Specifically, the papers in this special issue helped outline a number of avenues for future research to clarify and solve this debate. For example, the role of participants’ beliefs about the other’s perspective may play an important role in the interpretation of the RAS phenomenon and future research will need to take this into consideration. For example, Langton [28], Wiese et al. [29], and Gardner et al. [21] pointed out that participants must believe that the directional cue represents an intentional agent in order to take its point of view. In this case, however, the shift of attention is not “reflexive” but is a voluntary, top-down, process.

In addition, the high level of individual variation needs to be accounted for in future work. For example, Prpic [43] showed that perceiving musical note values causes a spatial shift of attention in expert musicians but not in non-experts. Similarly, Bukowski and Samson [42] found individual differences in the ability to handle conflicting perspectives. 

Furthermore, the research in this issue distinguishes among attentional orienting, level 1, and level 2 perspective-taking [34]. It may be the case that social factors have differential effects on each of the previously mentioned processes. Therefore, it will be important going forward for researchers to be specific about which type of perspective-taking is under examination. Lastly, evidence from the current issue suggests that certain effects might depend on the cognitive demand of the experimental task [19,34,49], which indicates that social factors are involved when the task is cognitively demanding, while they may not be necessary in other cases. 

Additional contributions presented in this special issue move away from the social-perceptual debate by trying to provide further insight about the nature of the cues and other variables that may influence RAS and attentional cueing paradigms [27,31,41]. Among those, further confirmations that the cognitive demand of a task plays an important role in attentional orienting have been provided. Specifically, Albonico et al. [30] provided evidence that the deployment of focal attention depends on the interaction between the task demand and the type of the directional cues. 

In conclusion, the contributions to the special issue greatly improved our understanding of the RAS phenomenon, and opened up new avenues of investigation, which may allow for a deeper, more sophisticated interpretation of RAS, which may go beyond the perceptual versus social interpretations.
